# Assessing performance of augmented reality-based neurosurgical training

**DOI:** 10.1186/s42492-019-0015-8

**Published:** 2019-07-03

**Authors:** Wei-Xin Si, Xiang-Yun Liao, Yin-Ling Qian, Hai-Tao Sun, Xiang-Dong Chen, Qiong Wang, Pheng Ann Heng

**Affiliations:** 1 Guangdong Provincial Key Laboratory of Computer Vision and Virtual Reality Technology, Shenzhen Institutes of Advanced Technology, Chinese Academy of Sciences, 1068 Xueyuan Avenue, Shenzhen University Town, Shenzhen, 518055 China; 2 Department of Neurosurgery, Zhujiang Hospital, Southern Medical University, Guangzhou, 510282 China; 3 E.N.T.department of Shenzhen University General Hospital, Shenzhen, 518055 China; 4 Department of Computer Science and Engineering, the Chinese University of Hong Kong, Hong Kong, China

**Keywords:** Augmented reality, Personalized virtual operative anatomy, Neurosurgical training

## Abstract

This paper presents a novel augmented reality (AR)-based neurosurgical training simulator which provides a very natural way for surgeons to learn neurosurgical skills. Surgical simulation with bimanual haptic interaction is integrated in this work to provide a simulated environment for users to achieve holographic guidance for pre-operative training. To achieve the AR guidance, the simulator should precisely overlay the 3D anatomical information of the hidden target organs in the patients in real surgery. In this regard, the patient-specific anatomy structures are reconstructed from segmented brain magnetic resonance imaging. We propose a registration method for precise mapping of the virtual and real information. In addition, the simulator provides bimanual haptic interaction in a holographic environment to mimic real brain tumor resection. In this study, we conduct AR-based guidance validation and a user study on the developed simulator, which demonstrate the high accuracy of our AR-based neurosurgery simulator, as well as the AR guidance mode’s potential to improve neurosurgery by simplifying the operation, reducing the difficulty of the operation, shortening the operation time, and increasing the precision of the operation.

## Introduction

Brain tumor resection is a routine treatment of brain tumors. During the surgery, surgeons first cut the skull open and then cut out the portions of the tumor that are accessible with a scalpel. To maintain quality outcomes, surgeons must undergo a series of neurosurgical training to acquire skills for procedures with varying degrees of complexity. However, traditional training activities are intensive in terms of both time and cost. Thus, practitioners turn to computer-assisted surgical training which can provide an alternative training mode with higher efficiency and at a lower cost.

In traditional neurosurgery, surgeons are guided by the pre-operative magnetic resonance (MR) images to approach the target tumor. However, this 2D image-based modality lacks the 3D structural guidance of the surrounding tissues. Also, users have to repeatedly switch the perspective between the surgical field and the MR images, which increases the difficulty of the operation and reduces the precision of the operation. By fusing imaging modalities with a real object, augmented reality (AR) can highly enhance the surgeons’ sensory experience by providing users with perception of depth and 3D spatial relationships [1]. Therefore, by integrating AR into neurosurgery guidance, surgeons can directly observe the hidden anatomical information and the true surgical environment at the same time. This would greatly improve the efficiency and accuracy of surgery.

With increasing use of neurosurgery simulators [2, 3], some have been validated for use in practicing neurosurgery. The National Research Council Canada developed a virtual reality (VR) simulator, called NeuroTouch, to simulate neurosurgical procedures such as brain tumor resection, ventriculostomy, and endoscopic nasal navigation [4–6]. The finite element method is adopted to simulate basic physical phenomenon including brain deformation and tissue removal. Based on the physical simulation, the simulator can assess tissue removal and bleeding in virtual surgeries. They developed the full virtual environment where patient-specific anatomy, complex physiological behaviors and elaborated performance metrics can be simulated. However, due to the limitation of visualization, users of the VR-based training system are completely immersed in the virtual environment without cognition of reality. In contrast, AR systems bring components of the virtual world into a person’s perception of the real world and provide higher fidelity than VR systems. Moreover, the “see-through” display mode in AR systems enables users to interact more naturally with both the real surgical field and the surgical simulator, and to coordinate their vision and the operation. Besides, the VR-based simulator usually represents the surgical tools as two shafts in a box. This is very different to real surgery. Meanwhile, an AR-based simulator can combine the real and virtual objects with stereo tracking technology.

This greatly improves immersion into the surgical environment and the accuracy of perception of the operation region. Considering that a neurosurgery simulator can offer numerous benefits to users, including gaining experience free from risk to patients, learning from mistakes and rehearsal of complex cases [7], we thus employ neurosurgery simulation to explore the suitability of an AR technique in the guidance of neurosurgery.

To achieve immersive neurosurgery training with high fidelity, we have developed an AR-based neurosurgery training simulator, enabling surgeons to learn brain tumor resection manipulation skills in a more natural and intuitive way. In our system, accurate 3D personalized brain anatomy is segmented and reconstructed via deep Voxelwise Residual Networks (VoxResNet) [8]. The 3D brain is precisely registered to the 3D-printed skull via the positioning of the markers and the calibration of the stereo tracking system and HoloLens, thus enabling the holographic rendering of the 3D virtual brain overlaid on the real skull. To provide efficient simulation of the brain resection procedures, we modeled the mechanical behaviors of brain soft tissue using the mass-spring model and integrated bimanual haptic interaction to provide realistic haptic rendering of the surgical operation.

### Related work

In recent years, a number of simulators have been applied to achieve specific training objectives, such as the simulation technologies developed for intricate neurosurgical procedures [9, 10]. Phillips et al. [11] proposed the first neurosurgical VR simulator which is for ventricular catheter insertion. After a period of rapid development, a number of VR-based neurosurgical simulators have been developed for simulating cranial procedures [12].

Development of neurosurgical VR simulators was first reported to simulate ventricular catheter insertion but has developed rapidly [2, 11]. Several reports have also been published on VR simulation of neurosurgical endoscopic procedures [12]. A number of neurosurgical VR simulators have been developed for cranial procedures. VIVAN is the neurosurgical planning system in the VR environment Dextroscope [13]. It allows users to reach into a computer-generated stereoscopic 3D object and interact with the object with both hands behind a mirror. By integrating a high-fidelity haptic feedback module, the ImmersiveTouch system enables users to feel life-like resistance as they perform procedures and it has been validated for ventriculostomy [14, 15].

During an intracranial tumor resection, the goal of the operator is to resect the brain tumor with instruments using techniques and applying forces that adequately remove the brain tumor but result in minimal injury to surrounding normal brain tissue. However, the technical and cognitive aspects necessary to accomplish this goal by the expert neurosurgeon are not totally understood [2]. It is necessary to develop a system that can assess surgical operation skills. The NeuroTouch platform provides specific assessment of surgical operation skills during a simulated brain tumor resection, measuring the following outcomes: percentage of brain tumor resected, volume of simulated “normal” brain tissue surrounding the tumor removed, duration of time taken to resect the brain tumor, instrument path length, pedal activation frequency, and sum of applied forces [16].

Recently, on pace with the great development of AR technologies, AR-based surgical simulation has been widely explored for better surgical training and navigation. AR aligns and then seamlessly combines computer-generated virtual objects and the real environment to provide more immersive and realistic visual effects [17]. Even with these advantages, developing an AR-based surgical navigation system is a challenging task. Foremost among these challenges is overlaying virtual CT data onto a real patient’s anatomy [18]. Liao et al. [19] proposed an MRI-guided navigation system with auto-stereoscopic images. The system fixes a 3D image in space which is irrelevant to viewer position. Navab et al. [20] developed an X-ray C-arm system equipped with a video camera. The system fused a direct video view of a patient’s elbow with the registered X-ray image of the humerus, radius, and ulna bones.

## Methods

**Fig. 1:**
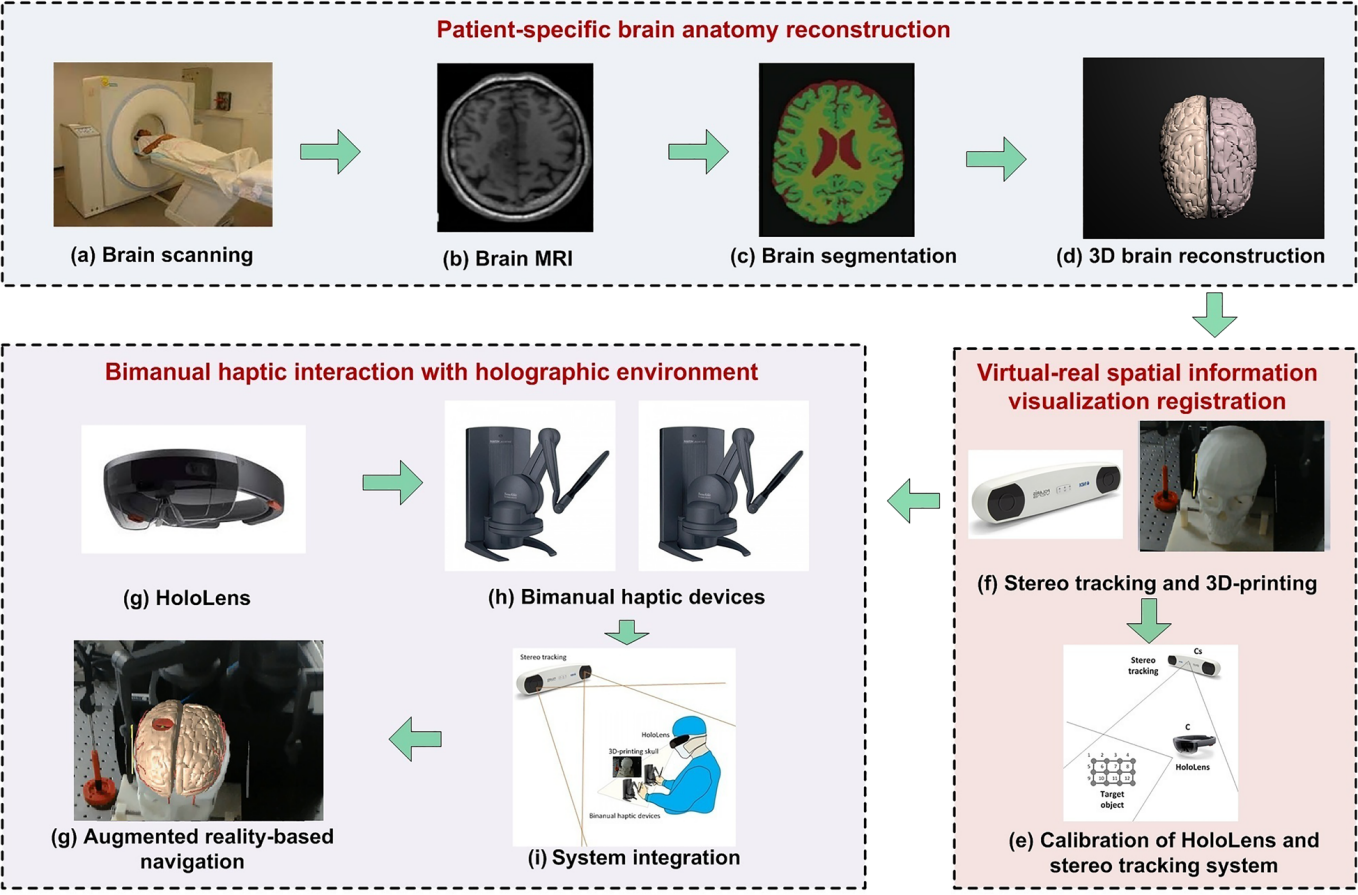
Overview of augmented reality guidance for neurosurgery training

In this paper, by introducing Microsoft HoloLens and Geomagic touch, we propose an AR-guided neurosurgical training simulator with haptic interaction for brain tumor resection procedures. Our ultimate goal is to integrate the AR guidance for neurosurgical training and assist users in accurately approaching the target tumors in neurosurgery procedures, making the surgery simpler, more efficient, and more accurate. The system consists of three components: patient-specific brain anatomy reconstruction, virtual-real spatial information visualization registration, and bimanual haptic interaction with a holographic environment. Figure 1 illustrates the overview of the developed system.

### Patient-specific anatomy reconstruction

**Fig. 2:**
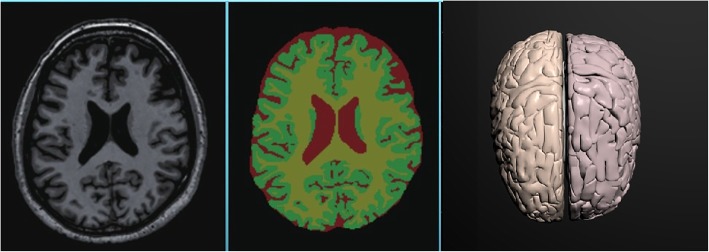
Brain segmentation and reconstruction

Anatomy reconstruction greatly influences the accuracy of neurosurgery simulation. Thus, to develop the AR guidance for neurosurgery, we first need to accurately reconstruct a 3D geometric model of the patient-specific brain, including the brain tissue, vessels, and tumors. MRI is the main choice for the diagnosis of brain disease. However, it is still a challenging task to accurately segment the brain and tumor with fine details from MR images. In this work, we adopt deep VoxResNet to precisely segment the brain MRI [8]. After segmentation, we adopt the mesh optimization approach based on newly developed bi-normal filtering [21] to removing noise while preserving the key anatomical features. As a result, a patient-specific virtual anatomic model with fine details can be reconstructed, as shown in Fig. 2.

### Mechanical interactions

Precise modeling of contacts in a surgical planning system is essential for obtaining a realistic global behavior of the deformable bodies during the interactively progressive cutting. Contact forces induced by tool−tissue and tissue−tissue interactions play the role of boundary conditions in the dynamic simulation and provoke the mechanical response of deformable bodies. Frictional contact problems are rather complicated from both theoretical and numerical points of view. They are characterized by a geometric and material discontinuity at the interface instead of the usual continuity property holding in solid mechanics. As a consequence, contact problems are inherently non-linear (even non-smooth), involving variational inequalities and constrained minimizations. Here we resolve the frictional contact problems of both tool−tissue and tissue−tissue interactions in surgical planning through Coulomb’s friction law with the Signorini condition [22] on our hybrid geometric model. We resolve the tool−tissue interaction with the penalty method, which is defined as the contact force **f**_*c*_,

1 $$ {\mathbf{f}}_c= k\delta \overrightarrow{n} $$

where *δ* is the interpenetration, contact normal $\overrightarrow{n}$ is equal to the surface normal of a triangle where the contact point is located, and $k=\frac{1}{h^2}\mathbf{M}+\frac{1}{h}\mathbf{D}+\mathbf{K}$, **M**, **D**, **K**, *h* are the mass, damping, stiffness matrix, and time step respectively, all of which can be precomputed before simulation.

### Biomechanical model for cutting and deformation

Brain tumor resection manipulations involve physical procedures, such as brain tissue deformation, cutting and bleeding. Accurately simulating these procedures is one of the most important steps in providing a realistic virtual surgical environment. The brain contains different types of structures (soft tissue, tumor, and vessels), which will definitely impact the biomechanical deformation of the brain. A mass-spring based soft tissue model is proposed to simulate the brain deformation. In the model, each kind of structure has a unique material property and different types of structures couple with each other, to better describe the biomechanical behavior of the brain with tumor and vessels. The soft tissue was discretized by a set of mass points and interconnected via a network of springs. The mass-spring model is well-suited for modelling an object with complex material properties such as nonlinearity and viscoelasticity. The following equation describes the dynamics of the mass-spring system:

2 $$ M\ddot{x}+D\dot{x}+ Kx=F $$

where $\ddot{x}$, $\dot{x}$ and *x* are the acceleration, velocity and position of the mass point, *M* is the mass, *D* is the damping coefficient, *K* is the elastic coefficient and *F* is the external force.

**Fig. 3:**
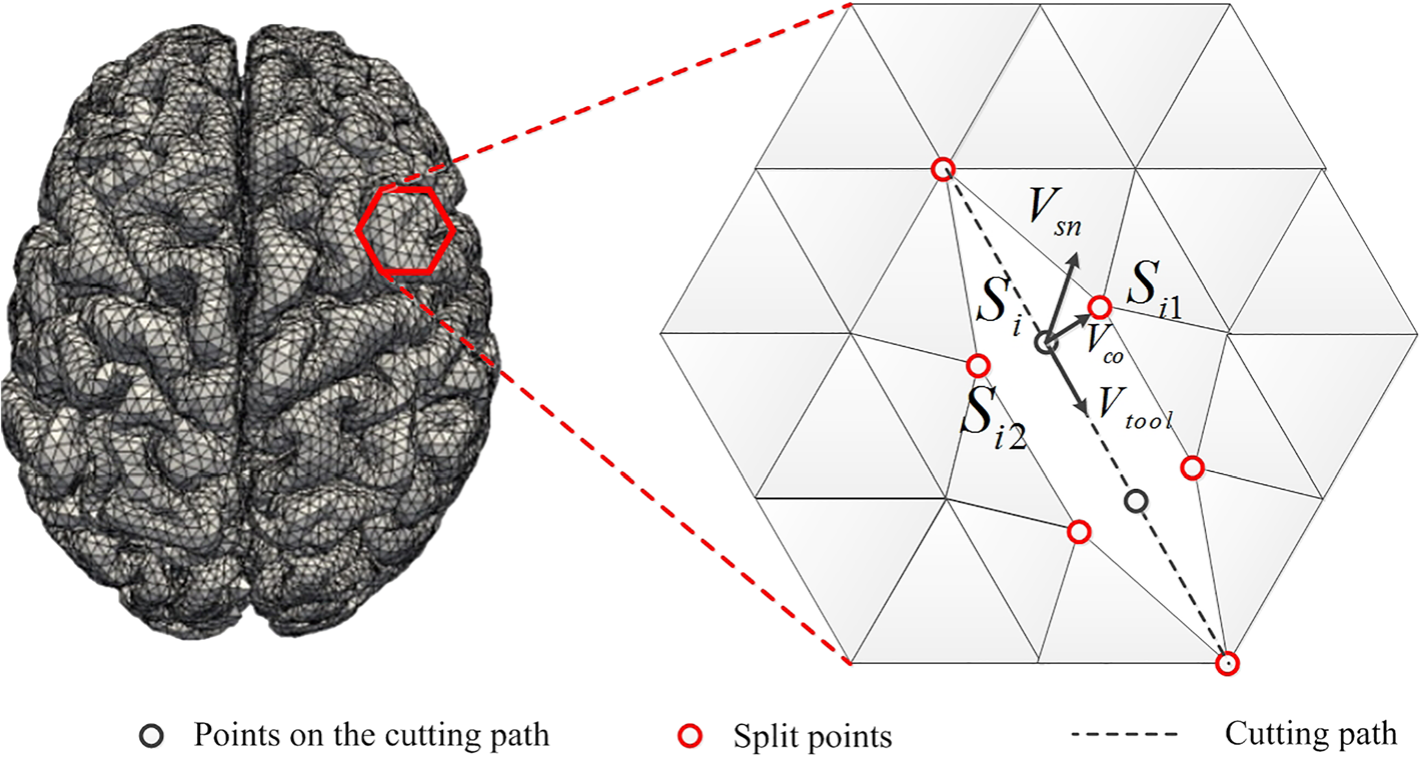
Cutting procedures

In this work, we also adopt the mass-spring model to simulate the accompanying deformation during the interactively progressive cutting procedure. During the cutting procedure, we first detect the collision points on the mesh object, then we deform the brain soft tissue with the mass-spring model, and split each point on the cutting path into two corresponding points when the contact force exceeds a threshold *f*_*threshold*_, dividing the cutting path into two separate parts with the method described in ref. [23]. As shown in Fig. 3, *S*_*i*_ is the mass point on the cutting path, which is duplicated twice and directly displaced at two sides of the cutting path such as vertex points *S*_*i*1_ and *S*_*i*2_ for *S*_*i*_. The displacement direction is perpendicular to the cutting path and the original mass point *S*_*i*_ is then deleted. All polygon edges connected to the deleted point *S*_*i*_ are reconnected to their duplicated points *S*_*i*1_ and *S*_*i*2_, which can be calculated by:

3 $$ {S}_{i1}={S}_i+\frac{W}{2}{V}_{co},{S}_{i2}={S}_i-\frac{W}{2}{V}_{co} $$

4 $$ {V}_{co}=\frac{1}{\left|{V}_{tool}\times {V}_{sn}\right|}{V}_{tool}\times {V}_{sn} $$

where *W* is cut opening width, *V*_*tool*_ and *V*_*sn*_ are the unit vectors along the tool’s direction of travel and the tangent plane normal at node *S*_*i*_, respectively.

**Fig. 4:**
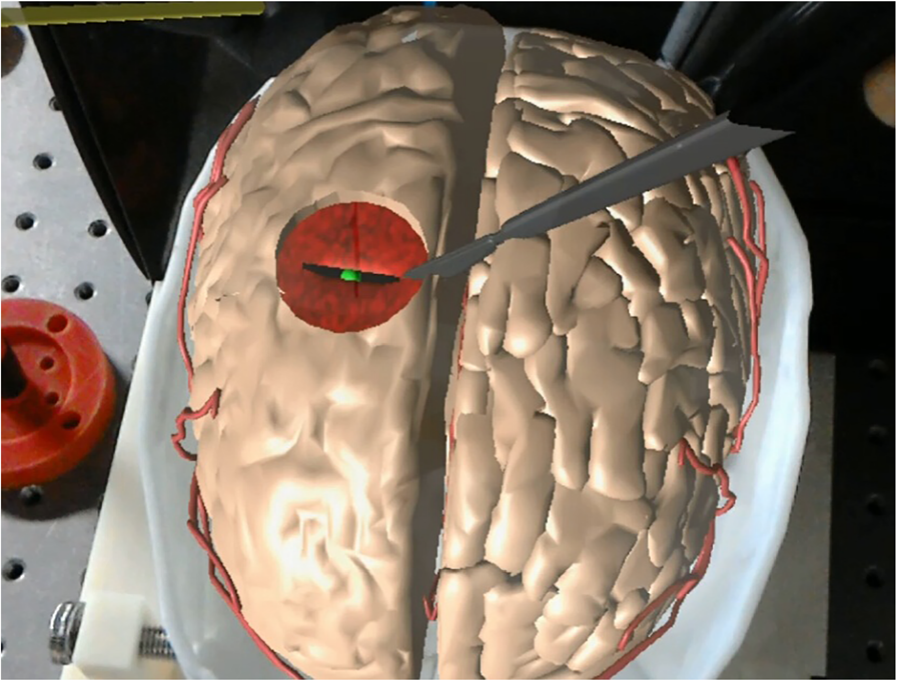
Biomechanical modeling of neurosurgery procedures

The simulated neurosurgery procedures are shown in Fig. 4.

### Virtual-real spatial information visualization registration

To achieve effective AR-based guidance, it is essential to register the virtual object in the real scene. Microsoft HoloLens is employed to overlay holographic brain anatomy onto a 3D-printed skull. With the “see-through” mode, the user can intuitively approach the target tumor as they would in a real operation. To achieve accurate mapping between virtual and real information, an interactive registration method is proposed to register the virtual skull model to the 3D-printed skull. Several non-coplanar points are selected on the printed model and correspondingly marked on the virtual skull model. Before the visualization registration, it is first necessary to calibrate the HoloLens in the NDI stereo tracking system. The steps are as follows:

- Step 1: Fix the marker of the NDI system on the HoloLens, and then acquire the position and orientation of the HoloLens equipment in the coordinate system of NDI, and calculate the angle *α*_*NDI*_ between its orientation and the NDI vertical direction.
- Step 2: With the required Y direction in the HoloLens coordinate system, it is possible to obtain the angle *α*_*H*_ between the Y axis and the NDI vertical direction. Then, it is possible to obtain the angle deviation between the HoloLens’s placement and its coordinate system, denoted as *α*_*H*_ − *α*_*NDI*_.
- Step 3: Set a virtual sphere in the HoloLens and set the origin of the HoloLens on this virtual sphere. By placing the virtual sphere on the origin of the NDI coordinate system, the origin of the HoloLens coordinate system and the NDI coordinate system are in the same place. Then, it is necessary to calibrate the angle between the Y axes of these two coordinate systems. The calibration matrix can be calculated by *T*(*α*_*H*_ − *α*_*NDI*_).
- Step 4: Given a position *C*_*s*_ in the NDI system, it is possible to calculate the corresponding position *C* in the HoloLens system by *C* = *T*(*α*_*H*_ − *α*_*NDI*_) ⋅ *C*_*s*_.

After the calibration of the HoloLens in the NDI stereo tracking system, it is possible to register and overlay the virtual skull into a real scene by tracking the positions of featured points on the skull model.

### Bimanual haptic interaction with a holographic environment

To achieve high fidelity guidance, both visual and haptic, we integrate bimanual haptic augmented interaction with two Geomagic touch devices. We develop our system with Unity3D and connect two touch devices using OpenHaptic toolkit, which provides the access and control for the two touch devices. With the UNET module of Unity3D, the desktop and HoloLens are treated as the server and client, respectively (see in Fig. 1i). Then, the system can obtain the movement of the two Geomagic touch devices, synchronize the position and orientation of the virtual surgical tools, and compute the haptic forces.

**Fig. 5:**
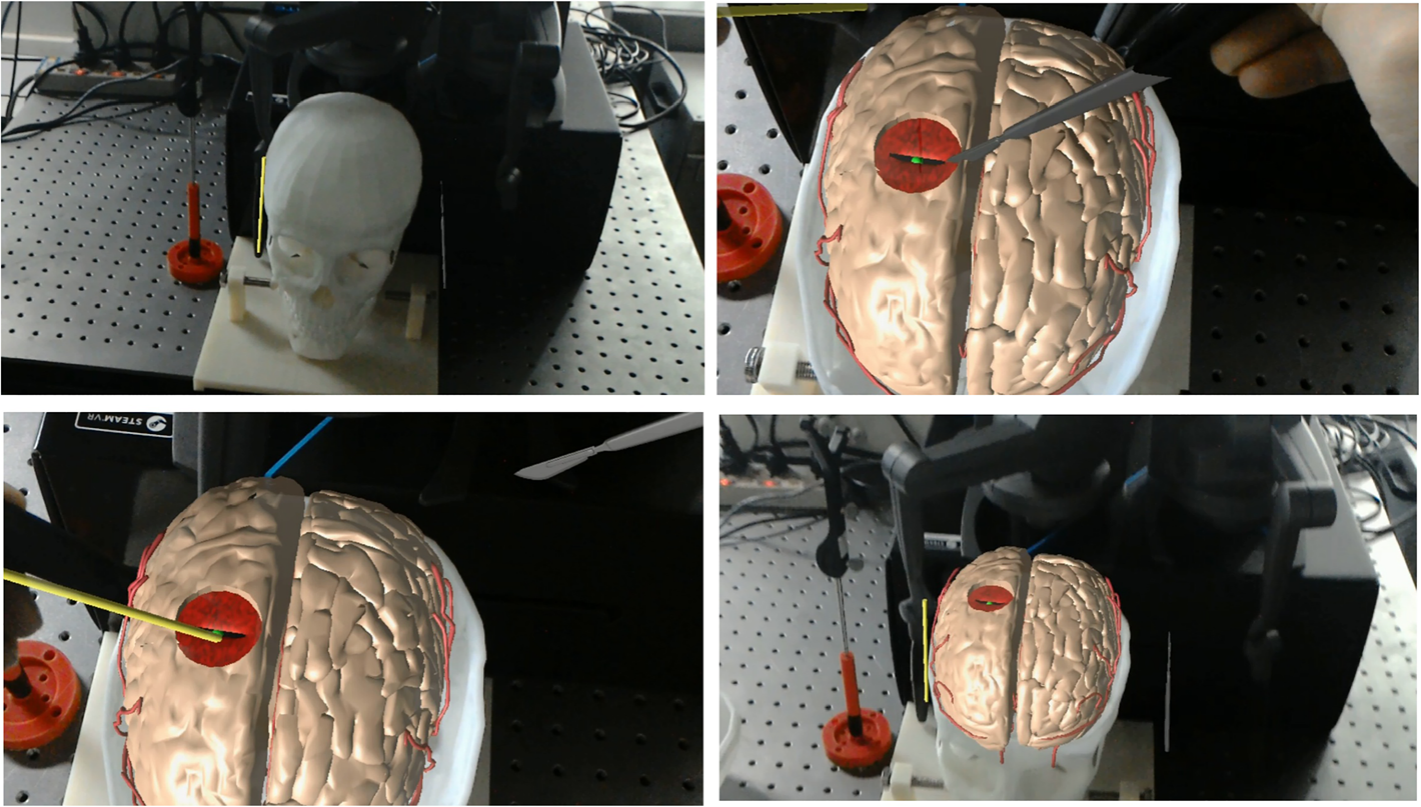
Augmented reality guidance for neurosurgery

To compute haptic forces, we detect the collision between virtual instruments and virtual brain with Signorini condition, and then compute the haptic forces via the penalty method [24]. Meanwhile, the selection of penalty coefficient is dependent on the stiffness ratio between the contacting or penetrating objects. The refresh rate of visual feedback in surgical simulation typically ranges between 25 and 60 Hz (vision rendering rates), while haptic feedback should run at a faster rate (1000 Hz). Here, we introduce the multirate technique to render the haptic feedback. In more detail, the dynamic deformation of the brain soft tissue is computed with the mass-spring model, while the feedback forces induced by tool−tissue interaction are solved with the penalty method, which is recomputed at a higher rate in the haptic loop to render the haptic feedback smoothly. To achieve more smooth and realistic haptic feedback, we adopt a B-spline linear interpolation to smooth the force output to increase its quality of continuity. With integrated bimanual haptic feedback, the developed AR-based neurosurgery training simulator is shown in Fig. 5.

## Results

We assess the performance of the AR-based neurosurgical training simulator and conduct a user study to evaluate the face and content validity. All experiments are conducted on a PC equipped with Intel(R) Xeon(R) E5–2640 v3 CPU (2.60GHz), 64G RAM, and NVIDIA GeForce GTX TAITAN.

### Neurosurgery simulation

We perform the neurosurgery operation with the AR guidance to test the AR guidance mode (Fig. 5). With the brain anatomy reconstruction algorithm, we obtain the 3D polygon mesh of the brain which consists of 29,984 triangles and 14,733 vertices. By registering the reconstructed brain anatomy to the 3D-printed skull, we are able to clearly observe the hidden anatomical structures of the brain, including target tumors, vessels, and brain soft tissue, which greatly facilitate the brain tumor resection operation, making the operation much easier. Moreover, natural interactions are also enabled in our simulator via the “see-through” display, which benefits the users by coordinating their vision and operation, and thus increasing the precision of brain tumor resection.

### Accuracy validation

**Fig. 6:**
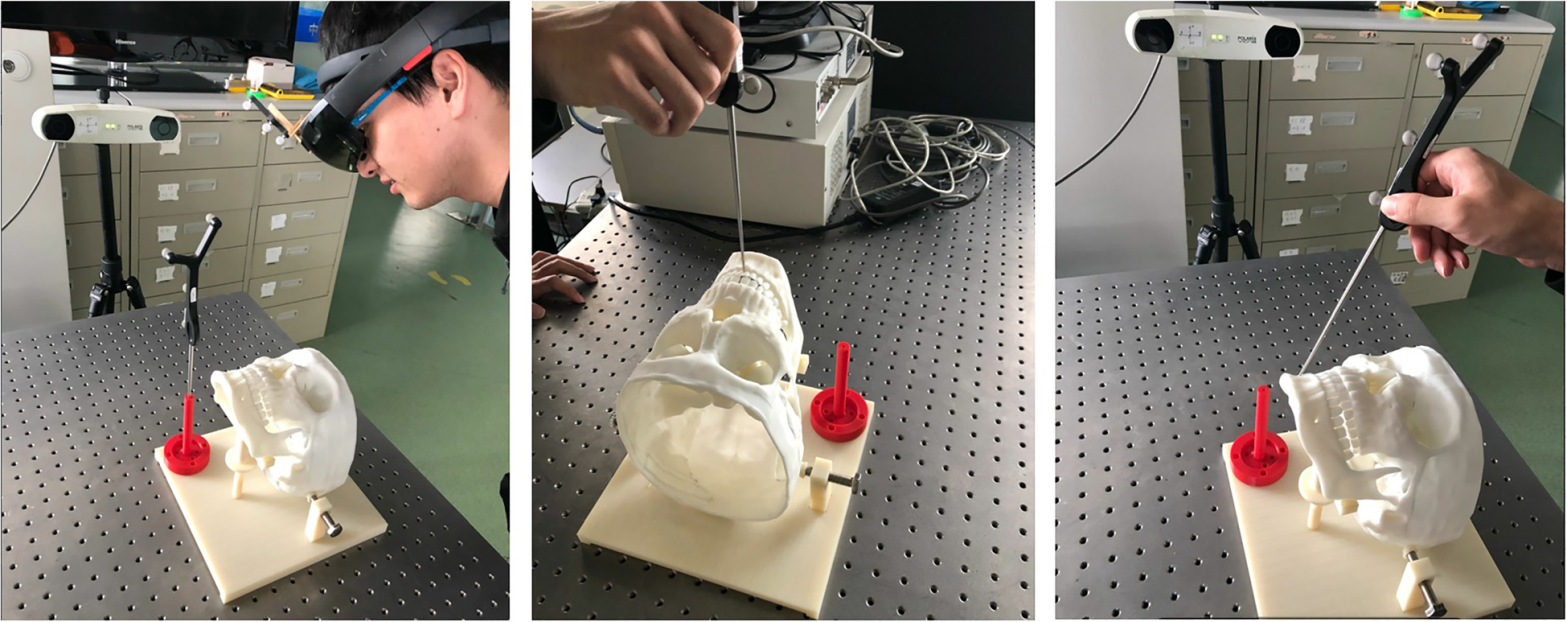
Accuracy validation of the augmented reality-based neurosurgery navigation

We also design an accurate 3D-printed template and skull to perform the accuracy validation experiment, to determine the accuracy variation of our AR guidance. Figure 6 shows a verification experiment in this work. We have reconstructed the virtual skull based on the personalized CT/MR images. The 3D-printed template and skull was designed with several markers, which lie at a standard distance with high precision (0.1 mm), that were used for measuring the distance error and angular error of the AR-based navigation.

In this experiment, we validate all procedures during the personalized augmented reality-based neurosurgery, such as the tracking and positioning of the surgical instruments, real and virtual scene registration, and surgical operation. First, we put the 3D-printed skull and template on the tracking area of the 3D tracking and positioning system, then we calibrate the positioning of surgical tools through the calibration template with markers collected on the 3D-printed skull and the virtual 3D skull model. Based on the real and virtual scene registration by the HoloLens, the position of the markers on the 3D-printed skull and the virtual skull can be obtained. Here we check if the virtual skull model is aligned with the 3D-printed skull, and we calculate the relative position of the markers both by the tracking system and the virtual markers on the virtual skull, which were used to compare the distance error to validate the accuracy of our system. Suppose the positions of the markers on the 3D-printed skull and template are *C*_1_, *C*_2_, ⋯, *C*_*n*_, the calculated positions of these markers with our method are *C*_1_ ' , *C*_2_ ' , ⋯, *C*_*n*_', and therefore the registration error can be computed as:

5 $$ TRE=\sqrt{\frac{\sum \left\Vert {C}_i-{C}_i\hbox{'}\right\Vert }{n}},i=1,2,\cdots n $$

In the experiment, 10 feature points are selected for registration error evaluation. The real position and the registration position of all feature points are as shown in Table 1. Results show that the average target registration error of the proposed registration algorithm is 2.11 mm.

**Table 1:** Performance statistics of automatic registration

Markers	Real position (mm)	Registered position (mm)	Displacement (mm)	Registration error (mm)
1	(57.31,-81.97,-808.93)	(58.12,-82.19,-809.26)	(−0.81,0.22,0.33)	0.90
2	(54.25,-61.99,-808.53)	(54.53,-63.01,-807.87)	(−0.28,1.02, − 0.66)	1.25
3	(60.05,-40.90,-810.51)	(60.21,-40.72,-811.14)	(−0.16,-0.18, 0.63)	0.67
4	(57.52,-84.09,-836.63)	(60.80,-86.32,-835.86)	(−3.28,2.23, −0.77)	4.04
5	(62.65,-39.85,-834.71)	(60.78,-40.03,-835.77)	(1.87, 0.18, 1.06)	2.16
6	(41.02,-65.90,-880.98)	(41.59,-67.40,-880.14)	(−0.57,1.50, − 0.84)	1.81
7	(84.00,-119.77,-902.75)	(84.02,-122.13,-904.07)	(−0.02, 2.36, 1.32)	2.70
8	(43.72,-89.05,-895.05)	(44.63,-90.15,-894.14)	(−0.91,1.10, − 0.91)	1.69
9	(45.77,-42.38,-895.80)	(46.54,-43.32,-896.44)	(−0.77, 0.94, 0.64)	1.37
10	(35.41,-68.06,-907.00)	(33.64,-67.30,-908.25)	(1.77, −0.76, 1.25)	2.30

### User study

We have designed a user study to assess the face and content validity of our AR-based neurosurgical training simulator. The questionnaire is designed according to a discussion with an expert in neurosurgery, to assess the effect of the AR guidance and the similarity between the simulated neurosurgery and real neurosurgery. We recruited 10 participants without any experience in real neurosurgery. They already knew the surgical scene of brain deformation and cutting from previous viewing of a teaching video of neurosurgery, and they had used surgical instruments to cut the soft tissue of animals. The questions about likeness to reality, “realistic,” for the AR environment mainly assess the similarity between the AR-based neurosurgical training simulator and the real neurosurgery shown in the teaching video. The questions about “realistic” for the virtual surgical instruments and haptic operation mainly assess the similarity between the feeling of using a haptic device and the feeling of using real surgical instruments. We prepared a technical instruction sheet outlining a goal and the operation steps of the AR-based neurosurgery training simulators. Participants were given 10 min to experience the manipulation of the equipment. Then, participants were required to complete a neurosurgery procedures six times. They were required to find the tumor inside the brain tissue and complete the brain tumor resection operation. Face and content validity were evaluated by asking participants to complete a questionnaire after the study. They were asked to respond to statements using “agree,” “neutral,” and “disagree.”

For face validity, participants were asked to evaluate the following statements related to the behavior of the real and virtual instruments and their interactions with the brain soft tissue:

- Q1: The brain soft tissue in the AR-based environment was realistic.
- Q2: The tumor inside the brain soft tissue in the AR-based environment was realistic.
- Q3: The AR-based brain tissue and tumor inside the 3D-printed skull was realistic.
- Q4: The 3D-printed skull was realistic.
- Q5: The brain soft tissue deformation in the AR-based environment was realistic.
- Q6: The cutting of brain soft tissue in the AR-based environment was realistic.
- Q7: The interaction between virtual surgical tools and the virtual brain tissue was realistic.
- Q8: The interaction between brain soft tissue and the tumor was realistic.
- Q9: The positioning of the haptic device was accurate.
- Q10: The real-time registration between the virtual surgical tool and the haptic device was accurate.
- Q11: The feeling of operating the haptic device was like operating with surgical instruments.
- Q12: The delay between physical manipulation and visual reaction was realistic.
- Q13: The haptic force feedback felt realistic.

**Fig. 7:**
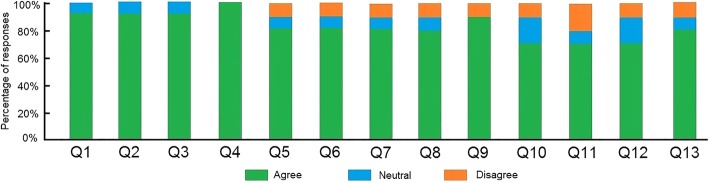
Face validation results

Figure 7 shows the face validation results of this questionnaire for all participants. For the visualization of brain soft tissue and tumor in the AR environment (Q1-Q3), 90% of participants responded “agree”, confirming that the AR visualization is realistic, while 10% of the responses were “neutral”. All participants agreed that the 3D-printed skull is realistic (Q4). In terms of questions assessing the physical behavior of brain soft tissue and interaction in neurosurgery simulation (Q5-Q8), 80% of responses were “agree”, 10% were “neutral”, and 10% were “disagree”. For the questions assessing the performance of the AR-based navigation system (Q9-Q13), 90% of responses were “agree”, confirming that the positioning of the haptic device was accurate (Q9), while 10% were “disagree”; 70% of responses were “agree”, confirming that the registration between the virtual surgical tool and the haptic device was in real-time and accurate (Q10), while 20% were “neutral”, and 10% were “disagree”; 70% of responses were “agree”, confirming that the feeling of operating the haptic device was like operating with surgical instruments (Q11), 10% were “neutral”, and 20% were “disagree”; 70% of responses were “agree”, confirming that the delay between physical manipulation and visual reaction was realistic (Q12), while 20% were “neutral” and 10% were “disagree”; 80% of responses were “agree” the haptic force feedback felt realistic (Q13), while 10% were “neutral” and 10% were “disagree”. For content validation, participants were asked to evaluate the following statements to assess the adequacy of the simulated tasks and perceived utility of the simulator as a training tool for neurosurgery.

- Q1: The AR-based brain tissue and tumor inside the 3D-printed skull is sufficient to provide an immersive environment for training of neurosurgery.
- Q2: The 3D-printed skull is sufficient to make it a useful training tool for neurosurgery.
- Q3: The brain soft tissue deformation behavior is sufficient to make it a useful training tool for neurosurgery.
- Q4: The brain soft tissue cutting behavior is sufficient to make it a useful training tool for neurosurgery.
- Q5: The interaction between the virtual surgical tool and brain soft tissue and tumor is sufficient to make it a useful training tool for neurosurgery.
- Q6: The positioning and delay of the haptic device is sufficient to make it a useful training tool for neurosurgery.
- Q7: The real-time registration between the virtual surgical tool and the haptic device is sufficient to make it a useful training tool for neurosurgery.
- Q8: Overall the simulator is a useful training tool for neurosurgery.

**Fig. 8:**
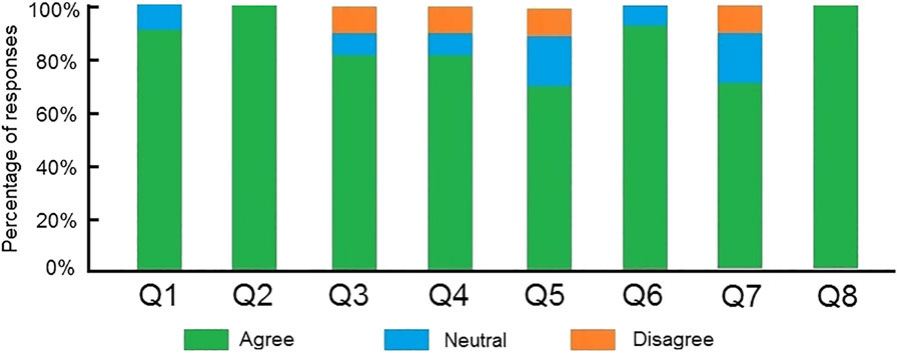
Content validation results

Figure 8 shows the content validation results of these questions for all participants. Ninety percent of participants responded with “agree”, confirming that the AR-based brain tissue and tumor inside the 3D-printed skull is sufficient to provide an immersive environment for neurosurgery training (Q1), while 10% of responses were “neutral”. All participants agreed that the 3D-printed skull is a useful tool for surgical training (Q2). For the questions assessing the effectiveness of the physical deformation and cutting simulation (Q3-Q4), 70% of responses were “agree”, confirming that the cutting and deformation is sufficient to make it a useful training tool for neurosurgery (Q3-Q4), while 10% were “neutral” and 10% were “disagree”. Sixty percentof responses were “agree” confirming that the interaction between the virtual surgical tool and brain soft tissue and tumor is sufficient for neurosurgery training (Q5), while 20% were “neutral” and 10% were “disagree”. For the AR system performance assessment, 90% of responses were “agree”, confirming that the positioning and delay of the haptic device is sufficient (Q6), while 10% were “neutral”. Ninety percentof responses were “agree”, confirming that the real-time registration between the virtual surgical tool and the haptic device is sufficient (Q7), while 20% were “neutral” and 10% were “disagree”. All the participants responded with “agree” to the statement that overall the simulator is a useful training tool for neurosurgery (Q8).

## Discussion

Neurosurgery is the most direct and effective method for the treatment of various brain diseases, but it is also one of the most difficult operations. It requires surgeons to resect the lesion accurately, while avoiding causing damage to normal tissues such as peripheral nerves and blood vessels. The success of neurosurgery depends largely on the accuracy of preoperative surgical planning and the accuracy of intraoperative lesion location.

Surgeons traditionally employ the guidance information provided by the preoperative MR images to approach the target tumor. However, the current guiding modality has two drawbacks. Firstly, the preoperative guidance information is separated from the intraoperative surgical scene. In order to observe the relative position between surgical instruments and the patient’s anatomical structure, surgeons have to switch the field of vision back and forth between the patient’s surgical scene and MR images, which is not desirable as it may affect the focus of surgeons. This will interfere with the surgical process and increase guidance error. Secondly, it is difficult for surgeons to understand the guidance information in the preoperative 2D MR images, which cannot accurately reflect the current position of surgical instruments in 3D space. Thus, surgeons cannot intuitively understand the spatial position relationship between the surgical instrument and the actual patient’s anatomical structure in the surgical scene, resulting in the inadequacy of the current guidance modality.

AR has certain advantages in the surgical guidance of neurosurgery. AR provides surgeons with accurate personalized anatomy structural information, which enables them to accurately assess the relative position between different structures. With the accurate representation and quantitative evaluation of anatomy structure, it is convenient for surgeons to determine the best access approach to the tumor. Most importantly, the AR enables surgeons to intuitively observe the internal structure by registering the reconstructed anatomy structure with the surgical scene, which provides clear guidance information for conducting the operation. With the developed AR-based neurosurgical training simulator, surgeons can experience realistic surgical operations comparable to real neurosurgery. However, the developed system has several limitations. Real neurosurgery has more details than the virtual scene of neurosurgery, such as the flowing of the blood and the pulsatility of blood vessels. In addition, the rendering of the AR environment needs to be improved to generate a surgical scene with high fidelity. By involving the detailed simulation of blood flow, vessel pulsatility and improving the rendering effects, the AR environment would be more accurate and immersive in the guidance of neurosurgery.

## Conclusions

In this paper, we explore the potential of AR technology being applied in neurosurgical training. Experiments show the effectiveness and accuracy of the developed neurosurgical training simulator, which can provide a very natural and intuitive way for surgeons to learn neurosurgical skills. However, our present study can only work on the simulated environment, where the registration problem can be handled with simple boundary conditions. In future work, we are to achieve the registration with sufficient accuracy in a real clinical scenario, thus enabling the AR-based visualization of target regions in surgical navigation. Meanwhile, we are also interested in modelling the details of blood flow, vessel pulsatility, and improving the rendering effects. In addition, extension to other guided surgeries is also a part of the further work.

## Data Availability

The datasets used and/or analyzed during the current study are available from the corresponding author on reasonable request.
